# Avaliação da normalidade dos dados em estudos clínicos e experimentais

**DOI:** 10.1590/1677-5449.041117

**Published:** 2017

**Authors:** Hélio Amante Miot

**Affiliations:** 1 Universidade Estadual Paulista – UNESP, Faculdade de Medicina de Botucatu, Departamento de Dermatologia e Radioterapia, Botucatu, SP, Brazil.

**Figura 1:**
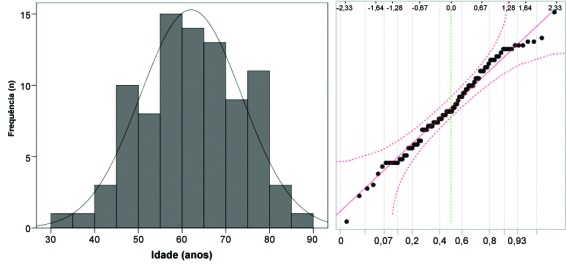
Pacientes (n = 89) portadores de úlceras venosas tratados no Serviço de Dermatologia da Faculdade de Medicina de Botucatu, Universidade Estadual Paulista (UNESP): histograma e diagrama Q-Q da idade em anos.

Os eventos naturais representados por dados contínuos assumem diferentes distribuições de frequência, entre elas uma distribuição em forma de sino, chamada curva normal ou de Gauss (Figura 1). A curva normal apresenta propriedades que a tornam especiais para a estatística, especialmente sua simetria, única moda (coincidente com a média e a mediana), além da possibilidade de ser representada e quantificada a partir dos valores da média e desvio padrão^1^.

Os principais testes estatísticos empregados na análise de dados clínicos e experimentais são baseados em modelos teóricos que pressupõem a distribuição normal, como teste *t* de Student, ANOVA, coeficiente de Pearson, regressão linear (resíduos) e análise discriminante^2^. Diante disso, a avaliação da normalidade da distribuição dos dados é primordial para a adequada descrição da amostra e sua análise inferencial^3^. Cálculos de tamanho amostral também são influenciados pela distribuição subjacente dos dados^4^.

**Figura 2:**
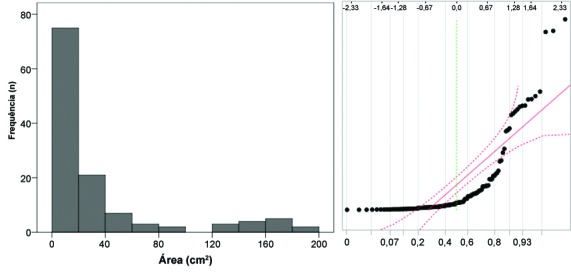
Úlceras venosas (n = 125) de pacientes tratados no Serviço de Dermatologia da Faculdade de Medicina de Botucatu, Universidade Estadual Paulista (UNESP): histograma e diagrama Q-Q das áreas em cm^2^.

Muitos dados biomédicos apresentam distribuição não normal, especialmente em eventos de grande variabilidade, com desvio padrão maior que a metade do valor médio (Figura 2), contraindicando o uso de técnicas estatísticas destinadas a amostras normais, sob pena de enviesamento dos parâmetros e da inferência dos testes^2^^,^ 5. Mesmo o aumento do tamanho amostral não suplanta os erros de estimativas causados pelo uso de distribuições inadequadas às técnicas de análise.

O primeiro passo para a avaliação da normalidade de um conjunto de dados deve ser a visualização de seu histograma, a fim de identificar grandes assimetrias, descontinuidades de dados e picos multimodais. É importante salientar que, na análise de subgrupos ou em comparações múltiplas, todas as categorias ou subamostras sujeitas à análise devem ser submetidas à avaliação da normalidade, e não apenas a amostra global^2^^,^ 3.

A Figura 1 exemplifica um histograma de dados que se aproxima da distribuição normal, enquanto a Figura 2 demonstra um histograma assimétrico, que se aproxima de uma distribuição de dados tipo gama.

Desde que o histograma não apresente inconsistências com a distribuição normal, é recomendável a avaliação dos estimadores de simetria e curtose, que representam aspectos ligados à forma do histograma: desviado para a esquerda/direita (simetria) ou apiculado/achatado (curtose); ambas as medidas se aproximam do zero quando os dados são normais. Como esses estimadores sofrem efeito do tamanho amostral e de valores extremos, é prudente calcular a razão de seus valores pelo erro padrão de suas estimativas. De forma geral, o valor do coeficiente dividido pelo seu erro padrão deve estar entre -1,96 e +1,96 nas distribuições normais^6^.

A Tabela 1 apresenta os valores de tendência central, dispersão, curtose e simetria das distribuições relativas às Figuras 1 e 2. Pode-se observar que, para as áreas das úlceras, tanto os valores de simetria como de curtose se distanciam do zero e, quando divididos pelos seus erros padrão, resultam em valores maiores que 1,96: 10,5 e 12,0.

**Tabela 1:** Estimadores de tendência central, dispersão e alguns testes de normalidade ligados à idade e à área das 125 úlceras venosas de 89 pacientes tratados no Serviço Dermatologia da Faculdade de Medicina de Botucatu, Universidade Estadual Paulista (UNESP).

	Idade (anos)	Área da úlcera (cm^2^)
Média (desvio padrão)	62,1 (11,6)	39,3 (62,9)
Mediana (p25-p75)	60,6 (53,0-71,8)	11,4 (4,0-38,4)
Curtose (erro padrão)	-0,50 (0,51)	5,14 (0,43)
Simetria (erro padrão)	-0,14 (0,26)	2,30 (0,22)
Teste D'Agostino-Pearson (p-valor)	1,41 (0,50)	55,52 (<0,01)
Teste de Lilliefors (p-valor)	0,05 (0,66)	0,27 (<0,01)
Teste de Shapiro-Wilk (p-valor)	0,99 (0,71)	0,67 (<0,01)

Diagramas quantil-quantil (diagramas Q-Q) são representações gráficas das proporções dos dados da amostra original em comparação com os quantis esperados para uma distribuição normal (Figuras 1 e 2). Nesses casos, o diagrama Q-Q deve, idealmente, se apresentar como uma linha diagonal caso os dados sejam próximos à distribuição normal. A mesma análise pode ser conduzida por diagramas P-P, em que a distribuição dos dados observados é comparada com o percentil cumulativo esperado de uma distribuição normal. Há uma tolerância para pequenos desvios que ocorrem nos valores mais extremos, como está representado pelas linhas de erro tracejadas na Figura 1. De forma geral, análises da normalidade baseadas nos diagramas Q-Q são as mais confiáveis para amostras de grande dimensão (> 5.000 unidades), quando os testes de normalidade inflacionam sobremaneira o erro tipo II (perdem sensibilidade)^7^^,^ 8.

Há uma dezena de testes estatísticos que verificam o ajuste dos dados à distribuição normal a partir de diferentes pressupostos e algoritmos. Todos os testes pressupõem a hipótese de normalidade dos dados (H0), retornando um p-valor > 0,05 se resultarem na aderência aos parâmetros de normalidade. Diversas simulações demonstram um melhor desempenho para os testes de Shapiro-Wilk e Shapiro-Francia^2^^,^ 9 ^-^ 14.

Os testes de normalidade sofrem influência do tamanho amostral quanto à sua eficiência. Em amostras pequenas (entre 4 e 30 unidades), há inflação do erro tipo I, sendo preferidos os testes de Shapiro-Wilk e Shapiro-Francia (maior especificidade). À medida que aumentam as amostras, especialmente acima de 500 unidades, todos os testes apresentam melhores desempenhos; entretanto, é prudente adotar o nível de significância de p < 0,01, em função do inflacionamento do erro tipo II causado pelo aumento amostral (perda de sensibilidade)^2^^,^ 11 ^,^ 14.

O teste de D'Agostino-Pearson foi desenvolvido para lidar com amostras mais numerosas (n > 100), apresentando, nesses casos, desempenho próximo ao do Shapiro-Wilk. O teste de Jarque-Bera apresenta bom desempenho na avaliação de normalidade em amostras maiores que 50 unidades, assim como o teste de Anderson-Darling^2^^,^ 12 ^,^ 13.

O teste de Kolmogorov-Smirnov deve ser dedicado apenas à verificação de aderência da amostra a distribuições com outros parâmetros, visto que é superado pelos outros aqui descritos para testar a normalidade dos dados. Por outro lado, o emprego da correção de Lilliefors oferece uma boa opção para analisar normalidade quando a distribuição contiver muitos dados extremos e a amostra for maior que 30 unidades^13^.

Dados que não se revelem aderentes à distribuição normal pelos métodos descritos anteriormente devem ser tratados com cautela pelos pesquisadores. Primeiramente, a descrição da amostra deve ser representada pelos quartis (mediana, p25 e p75), já que a média e o desvio padrão podem não reproduzir a tendência central e dispersão dos dados. Na Tabela 1, podemos observar a proximidade entre a média e a mediana na distribuição das idades dos pacientes (62,1 e 60,6 anos), assim como a discrepância que ocorre entre elas quando da representação das áreas das úlceras (39,3 e 11,4 cm^2^).

Há uma grande variedade de técnicas estatísticas destinadas a examinar amostras independentemente do formato de sua distribuição, que são chamadas estatísticas não paramétricas e englobam testes populares como Mann-Whitney, Wilcoxon, Kruskal-Wallis, Jonckheere-Terpstra, Friedman e coeficiente de Spearman. Essas técnicas substituem os dados originais por postos ordenados (*ranks*) de acordo com a escala de dados. De forma geral, esses testes apresentam maior erro tipo II, especialmente quando as amostras forem de menor dimensão (n < 30), além de tornarem menos generalizáveis as medidas de efeito^3^^,^ 14.

A transformação dos dados a fim de sua normalização é uma alternativa bastante usual em amostras com distribuição dos dados inclinada para a direita ou esquerda. Raiz quadrada, transformações logarítmicas, exponenciais, angulares (arcsen) e hiperbólicas (1/x) são as mais empregadas. Entretanto, deve-se ter em mente que, da mesma forma que as técnicas que empregam postos ordenados (*ranks*), as transformações dos dados alteram a escala entre as medidas, influenciando a interpretação direta e a generalização das medidas de efeito^15^.

Pode-se também optar por estratégias de análise de dados para distribuições especiais, como gama, uniforme, lognormal, beta, Tweedie, Poisson, binomial negativo, Weibull entre outras, chamadas: modelos lineares generalizados. Tais análises têm a vantagem de trabalhar com os valores (e a dimensão do efeito) na escala original; porém, pela maior complexidade dos processos analíticos, recomenda-se o auxílio de um profissional estatístico experiente^16^^-^ 18.

Em certas técnicas analíticas multivariadas (p.ex. MANOVA, análise de componentes principais e análise fatorial exploratória) ou em análise de medidas repetidas, há a necessidade de comprovação da normalidade multidimensional (esfericidade dos dados). Todavia, esse tópico transcende o escopo do texto^3^^,^ 19.

Finalmente, as estratégias de avaliação dos dados quanto ao ajuste à distribuição normal devem ser adequadamente descritas na metodologia, sendo essenciais ao sucesso do processo investigativo, além de refletir o cuidado do pesquisador com a análise dos dados, o que gera maior credibilidade aos resultados.
