# Supplementary material for: Avaliação da normalidade dos dados em estudos clínicos e experimentais
Source: J Vasc Bras. 2017 Apr-Jun;16(2):88–91. [Article in Portuguese] doi: 10.1590/1677-5449.041117 (PMC5915855; doi:10.1590/1677-5449.041117)
Supplement: Translation

# Translation: Assessing normality of data in clinical and experimental trials

**Published:** 2017

**Authors:** Hélio Amante Miot

**Affiliations:** 1 Universidade Estadual Paulista – UNESP, Faculdade de Medicina de Botucatu, Departamento de Dermatologia e Radioterapia, Botucatu, SP, Brazil.

When continuous data are used to represent natural events they can take a variety of different frequency distributions, one of which is a bell-shaped distribution that is known as the normal or Gaussian curve (Figure 1). Normal curves have properties that make them special from a statistical perspective, particularly their symmetry, their unique mode (which is the same as both the mean and the median), and the fact that they can be represented and quantified from the values of the mean and the standard deviation.^1^

**Figure 1:** Patients (n = 89) with venous ulcers treated at the Dermatology Service, Faculdade de Medicina de Botucatu, Universidade Estadual Paulista (UNESP), SP, Brazil: histogram and Q-Q plot for age in years.

The main statistical tests used for analysis of clinical and experimental data are based on theoretical models that assume a normal distribution, such as Student’s *t* test, ANOVA, Pearson’s coefficient, linear regression (residuals), and discriminant analysis.^2^ For this reason, testing data distributions for normality is an essential element of adequately describing samples and their inferential analysis.^3^ Sample size calculations are also influenced by the underlying data distribution.^4^

Many biomedical data have non-normal distributions, especially those representing events with great variability, with a standard deviation greater than half of the mean value (Figure 2); which contraindicates the use of statistical techniques appropriate for normal samples, which would risk introducing bias to parameters and to the inferences of tests.^2^^,^ 5 Even increasing the sample size cannot correct the estimation errors caused by using analytical techniques that are not suited to the data distribution.

**Figure 2:** Venous ulcers (n = 125) in patients treated at the Dermatology Service, Faculdade de Medicina de Botucatu, Universidade Estadual Paulista (UNESP), SP, Brazil: histogram and Q-Q plot for areas in cm^2^.

The first step in evaluating the normality of a dataset should be to examine its histogram to identify major asymmetries, discontinuity of data, and multimodal peaks. It is also important to stress that when analyzing subsets or conducting multiple comparisons, all of the categories or subsamples being analyzed must be tested for normality, and not just the overall sample.^2^^,^ 3

Figure 1 shows a histogram plotted from data that are approximated to the normal distribution, whereas Figure 2 shows an asymmetrical histogram, that are approximated to the gamma distribution.

Assuming that the histogram does not reveal elements that are not consistent with the normal distribution, it is then recommended that estimators of symmetry and kurtosis should be calculated. These represent elements related to the shape of the histogram, dislocation to the left/right (symmetry) or peaked/flattened shapes (kurtosis), and both these measures approach zero when data are normal. Since these estimators are affected by sample size and outliers, it is prudent to calculate the ratio of their values to the standard error of their estimates. In general, the result of dividing the value of the coefficient by its standard error should fall in the range -1.96 to +1.96 for normal distributions.^6^

Table 1 lists the values for central tendency, dispersion, kurtosis, and symmetry for the distributions illustrated in Figures 1 and 2. It can be observed that the values for symmetry and for kurtosis for the data on area of ulcers are both far from zero and dividing them by their standard errors produces values greater than 1.96: 10.5 and 12.0.

**Table 1:** Estimators of central tendency, dispersion, and certain tests of normality related to data for patient age and area of 125 venous ulcers in 89 patients treated at the Dermatology Service, Faculdade de Medicina de Botucatu, Universidade Estadual Paulista (UNESP), SP, Brazil.

	**Age (years)**	**Area of ulcer (cm^2^)**
Mean (standard deviation)	62.1 (11.6)	39.3 (62.9)
Median (p25-p75)	60.6 (53.0-71.8)	11.4 (4.0-38.4)
Kurtosis (standard error)	-0.50 (0.51)	5.14 (0.43)
Symmetry (standard error)	-0.14 (0.26)	2.30 (0.22)
D´Agostino-Pearson test (*p*-value)	1.41 (0.50)	55.52 (<0.01)
Lilliefors test (*p*-value)	0.05 (0.66)	0.27 (<0.01)
Shapiro-Wilk test (*p*-value)	0.99 (0.71)	0.67 (<0.01)

Quantile-quantile plots (Q-Q plots) are graphical illustrations of the proportions of the data from the original sample compared against the quantiles expected for a normal distribution (Figures 1 and 2). Ideally, the Q-Q plot should follow a diagonal line if the data distribution is close to normal. The same analysis can be conducted using P-P plots, in which the distribution of the observed data is compared with the cumulative percentile expected from a normal distribution. There is a tolerance for minor deviations that occur at the extremes, as illustrated by the error lines plotted in Figure 1. In general, analyses of normality based on Q-Q plots are more reliable for large-scale samples (> 5,000 units), when tests of normality can greatly inflate type II error (reducing sensitivity).^7^^,^ 8

There are dozens of statistical tests for verifying the fit of data to a normal distribution, based on different assumptions and using different algorithms. All of them test the null hypothesis (H0) that the data are normal, and so they return *p*-value > 0.05 if the result shows that data do fit the parameters for normality. Several simulations have demonstrated that the Shapiro-Wilk and Shapiro-Francia tests offer better performance.^2^^,^ 9 ^-^ 14

The efficacy of normality tests suffers influence from sample size. With small samples (from 4 to 30 units), type I error is inflated and the Shapiro-Wilk and Shapiro-Francia tests are preferable (for better specificity). As sample sizes increase, especially over 500 units, all of the tests offer better performance; however, it is prudent to adopt a significance level of p < 0.01, because of the inflation of type II error caused by larger samples (reducing sensitivity).^2^^,^ 11 ^,^ 14

The D´Agostino-Pearson test was developed to deal with larger samples (n > 100), in which case it offers similar performance to the Shapiro-Wilk test. The Jarque-Bera test offers good performance for evaluating normality in samples larger than 50 units, as does the Anderson-Darling test.^2^^,^ 12 ^,^ 13

The Kolmogorov-Smirnov test should be reserved for testing the fit of a sample to distributions with other parameters, since it is outperformed by the other tests mentioned here for testing the normality of data. On the other hand, using the Lilliefors correction is a good option for analyzing normality when the distribution contains many extreme data and the sample is larger than 30 units.^13^

Data that are proven not to fit the normal distribution using the methods described above should be treated with care by researchers. Initially, the sample should be described using quartiles (median, p25, and p75), since the mean and standard deviation may not reflect the central tendency and dispersion of the data. In Table 1, for example, it can be observed that the mean and median of the distribution of patients’ ages are similar (62.1 and 60.6 years), whereas there is a large discrepancy between the mean and median in the data for areas of ulcers (39.3 and 11.4 cm^2^).

There is a large number of statistical techniques for analyzing samples that are not dependent on their distribution. These are known as nonparametric statistical techniques and they include popular tests such as the Mann-Whitney, Wilcoxon, Kruskal-Wallis, Jonckheere-Terpstra, Friedman, and also Spearman coefficients. These techniques rely on substituting the original data with their ordered ranks, according to the scale of the data. In general, these tests are subject to greater type II error, especially when the samples are smaller (n < 30) and their measures of effect are less generalizable.^3^^,^ 14

One option that is widely used for samples with distributions shifted to the right or to the left is to perform a mathematic transformation to normalize them. Square roots, logarithmic, exponential, angular (arcsin), and hyperbolic (1/x) transformations are the most usually employed. However, it should be remembered that, in common with techniques that use rank ordering, data transformations alter the scale between measures, influencing interpretation and generalization of measures of effect.^15^

It is also possible to opt for strategies for analysis of data for specific distributions, such as gamma, uniform, log-normal, beta, Tweedie, Poisson, negative binomial, Weibull, and others, which are known as generalized linear models. These analyses offer the advantage of working with the values (and the dimension of the effect) in the original scale; but because of the greater complexity of the analytical processes involved, it is recommended that help is sought from an experienced professional statistician.^16^^-^ 18

For certain multivariate analytical techniques (for example, MANOVA, principal components analysis, and exploratory factor analysis) or in analyses of repeated measures, it is necessary to demonstrate multidimensional normality (sphericity of data). Nevertheless, this is beyond the scope of this editorial.^3^^,^ 19

Finally, strategies for assessment of the fit of data to the normal distribution must be adequately described in the methodology, since they are essential to the success of the investigative process, in addition to demonstrating the care researchers have taken with analysis of the data, conferring greater credibility on the results.
